# Single Step Purification of Novel Thermostable and Chelator Resistant Amylase from *Bacillus* *Licheniformis* RM44 by Affinity Chromatography

**Published:** 2017

**Authors:** Ayesha Siddiqui, Mustafa Kamal, Seyed Abdulmajid Ayatollahi, Mohsin Ali, Mansoor Ahmed

**Affiliations:** a *Department of Biotechnology, University of Karachi, Karachi-75270, Pakistan. *; b *Phytochemistry Research Center, Shahid Beheshti University of Medical Sciences, Tehran, Iran. *; c *Department of Pharmacognosy, School of Pharmacy, Shahid Beheshti University of Medical Sciences, Tehran, Iran. *; d *Department of Chemistry, University of Karachi, Karachi-75270, Pakistan. *; e *Department of Pharmaceutical Chemistry, University of Karachi, Karachi-75270, Pakistan.*

**Keywords:** Amylase, Affinity chromatography, Thermostable, Chelator resistant, SDS-resistant

## Abstract

*Bacillus licheniformis* RM44 was isolated from hot spring near Karachi and screened for the production of extracellular amylase Amy RM44. Amy RM44 was purified to homogeneity on a single step by affinity chromatography using insoluble corn starch. The molecular weight of Amy RM44 was estimated to be 66 kDa by SDS–PAGE and zymographic analysis. Nine fold purification was achieved with the specific activity of 870 U/mg that provides the total yield of the enzyme up to 31%. Studies on purified AmyRM44 characterization revealed that the optimum temperature of enzyme was 100 ºC. Amy RM44 was proved to be highly thermostable as it retained 50% activity after 2 h at 100 ºC. Amy RM44 was stable over wide range of pH with optimum activity at pH 5. Enzyme activity was not significantly inhibited by SDS and EDTA. Amy RM44 also exhibited its activity towards various carbohydrates such as dextrin, pullulan, α-cyclodextrin, β-cyclodextrin, and γ-cyclodextrin.

## Introduction

Production of commercially important enzymes from microorganisms is of great interest in present day biotechnology. Microbial enzymes are marketed worldwide and used in various industrial processes including synthesis of sugar syrups, bread, juices, paper sizing as well as in paints and textile products (1). Starch is a complex polymer of amylose and amylopectin and is a major daily utilized food ingredient. Several species of bacteria, actinomycetes and fungi secrete large amount of starch hydrolyzing enzymes in the culture medium which have many applications in biotechnological industries (2, 3). 

Amylases are the enzymes involved in the hydrolysis of starch and related polysaccharides. Amylases cover about 25% of world enzyme market and have a great utilization in fermentation, textile, food, and paper industries (4, 5). Purified enzyme with high specific activity at low cost is the basic requirement of the industries therefore research has been carried out to purify enzyme at single step to reduce the enzyme processing cost. Affinity chromatography has now replaced the conventional chromatography techniques and played a major role in downstream processing for the purification of desired enzyme to economize the process by single step purification (6, 7).

Therefore, the present study was focused on the isolation of new amylase producing *Bacillus* *sp.* and the purification of amylase by one step affinity chromatography to reduce the production cost. Further the characteristics of purified enzyme were evaluated which would be beneficial for starch processing industries.

## Experimental

*Isolation and identification of microorganism*

*Bacillus* *licheniformis *RM44 was isolated from the hotspring, Mangopir, and Karachi. The isolate was maintained on Luria starch agar slants. For the strain identification cultural, morphological and biochemical studies were performed according to the Bergey’s manual of bacteriology (8). Further, the strain was characterized on the basis of 16S rRNA sequencing (9).

*Culture condition for amylase production*

*Bacillus* *licheniformis *RM44 was cultured in Luria starch broth containg tryptone (10 g/L), sodium chloride (10 g/L), yeast extract (5 g/L), and soluble starch (10 g/L). pH of the medium was adjusted to 7.0. Culture was incubated at 37 ˚C in an orbital shaker for 72 h. After the completion of fermentation cells were harvested and cell free broth was used as a source of crude extracellular enzyme. Proteins were precipitated out by the gradual addition of 80% ammonium sulphate saturation in the cell free broth.

*Purification of amylase*

Protein precipitates were dissolved in 10 mL 0.1 M sodium phosphate buffer of pH 6.2 containing 0.5 M sodium chloride. The enzyme preparation was applied to insoluble corn starch column which was previously equilibrated with the same buffer. Unbound proteins from the corn starch column were washed out through the flow of buffer while bound proteins were eluted with a linear gradient of 0-2% dextrin solution. The collected fractions were applied on Luria starch agar plates and the amylase activity was determined by flooding the plates with iodine solution. Active fractions were pooled together and dialyzed overnight against 0.1 M sodium phosphate buffer of pH 6.2 to remove salt and dextrin (7).

*Amylase assay*

Amylase activity in the active fractions was determined at 80 ˚C for 10 min as described by Bernfeld (10) using 1% soluble starch dissolved in 0.1 M sodium phosphate buffer, pH 6.2. Reaction was stopped by the addition of dinitrosalicyclic acid and absorbance was recorded at 492 nm. One unit of amylase was defined as “the amount of enzyme that liberated 1 µmol of reducing sugar under specified conditions.”

*Protein estimation*

Protein concentration was estimated by the method of Bradford (11) using bovine serum albumin as standard.

*Polyacrylamide gel electrophoresis and Zymography*

Sodium dodecyl sulphate polyacrylamide gel electrophoresis was carried out as described by Laemmli (12) with 10% slab gels. Molecular weight of amylase was determined with reference to molecular weight markers. Active bands of amylase were visualized by Zymography under native and denaturing conditions (13).

*Effect of temperature and pH on enzyme activity*

Optimum temperature of enzyme was determined by performing the standard amylase assay at various temperatures ranging from 37-100 ˚C. Effect of pH was studied at optimum temperature using different buffers including sodium acetate buffer (pH 4.0-5.0), sodium phosphate buffer (pH 6.0-7.0), tris hydrochloric acid buffer (pH 8.0-9.0), and glycine sodium hydroxide buffer (pH 10.0-11.0). 

**Table 1:** Yield of Amy RM44 obtained through affinity chromatography

**Steps of purification**	**Total activity** **(U)**	**Total protein** **(mg)**	**Specific activity** **(U/mg)**	**Purification fold**	**Yield** **(%)**
Ammonium sulfate precipitates (80%)	10220	104	98	1	100
Affinity chromatography	3200	3.68	870	9	31

**Table 2:** Effect of activators and inhibitors on Amy RM44 activity

**Activators/Inhibitors (5mM)**	**Residual amylase activity (%)**
Control	100
NaCl	92
CaCl_2_	91
Na_2_O_3_S	91
HgCl_2_	35
HgCl_2_	50
EDTA	85
PMSF	75
SDS (1%)	44

**Table 3:** Amy RM44 activity with various substrates

**Substrates**	**Amylase activity (%)**
Starch	100
Pullulan	56
Dextrin	81
α-cyclodextrin	53
β-cyclodextrin	55
γ-cyclodextrin	54

**Figure 1:**
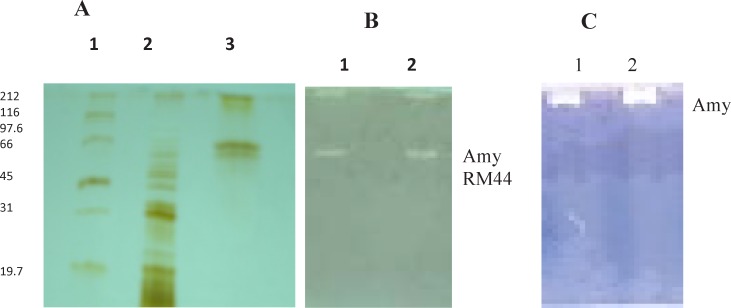
A) SDS-PAGE Lane 1: Molecular weight markers, Lane 2: 80% Ammonium sulphate precipitates, Lane 3: Purified enzyme from Affinity column. (B) Zymography Lane 1: 80% Ammonium sulphate precipitates, Lane 2: Purified enzyme from Affinity column. (C) Native PAGE Lane 1: 80% Ammonium sulphate precipitates, Lane 2: Purified enzyme from Affinity column

**Figure 2.**
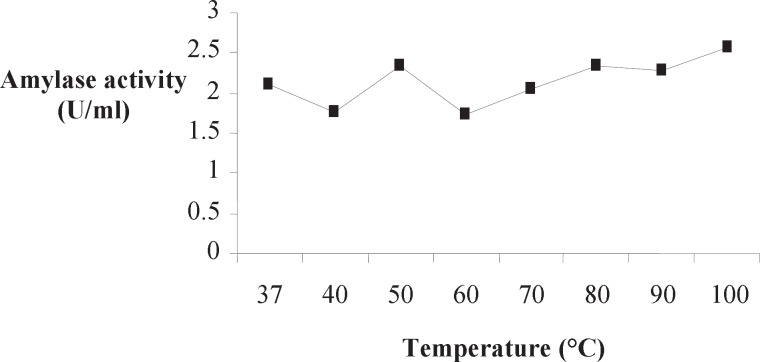
Effect of temperature on Amy RM44 activity

**Figure 3:**
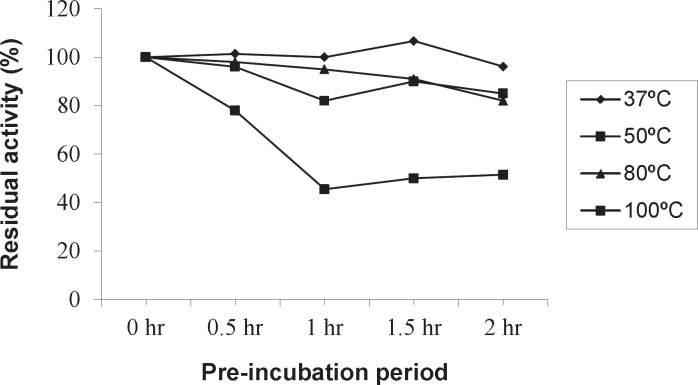
Thermostability of Amy RM44 at different temperatures

**Figure 4.**
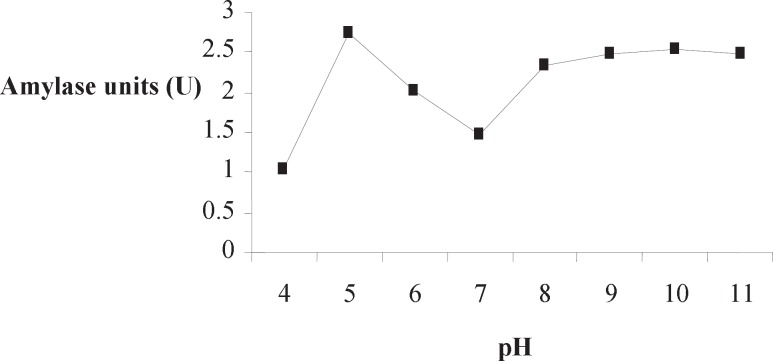
Effect of pH on Amy RM44 activity

*Thermal stability of enzyme*

Thermal stability of enzyme was determined at optimum temperature and pH by pre incubating the enzyme at 37, 50, 80, 100 ˚C for 2 h. Samples were collected after 30, 60, 90, and 120 min and assay was performed under standard conditions.

*Effect of activators and inhibitors on enzyme activity*

Activity of amylase in the presence of metal ions, chelating agent such as EDTA and inhibitors like SDS and PMSF was studied by pre-incubating the enzyme along with these reagents for 30 min. Assay was performed under standard conditions. Activity of amylase without metal ions and reagents was taken as 100%.

*Effect of substrates on enzyme activity*

Enzyme was mixed with 1% each of starch, dextrin, α-cyclodextrin, β-cyclodextrin, γ-cyclodextrin and pullulan. The amount of reducing sugars released was determined under standard assay procedure. Activity with starch was considered as 100%. 

## Results and Discussion

*Isolation and identification of amylase producing Bacillus licheniformis RM44*

Water sample from the hotspring of mangopir Karachi was collected and amylase producing bacteria were isolated. Among these cultures bacteriam RM44 was selected on the basis of high amylolytic activity on the plate test. Morphological and physiological characteristics showed that strain RM44 belonged to *Bacillus* genus as it was rod shaped, spore forming, gram positive and aerobic bacterium. Genomic DNA of *Bacillus* *sp.* RM44 was used for further characterization based on partial sequence obtained through 16S rRNA analysis. Sequence showed 99% homology with the 16S rRNA of *Bacillus licheniformis*. On the basis of obtained data the strain was named as *Bacillus licheniformis *RM44. Sequence was submitted in the gene bank having accession no. JN851867 (14).

*Amy RM44 production and purification*

*Bacillus licheniformis *RM44 was cultured for 72 h using 5% inoculums. Cell free broth was then used for the purification and characterization of amylase enzyme named Amy RM44. Amy RM44 was purified by affinity chromatography using insoluble corn starch as an absorbent matrix. Bound proteins were eluted by the linear gradient of 0-2% dextrin. Fractions containing Amy RM44 activity were obtained within 0.18-0.42% dextrin. Active fractions were pooled together for enzyme characterization. It has been shown that purified Amy RM44 possessed 870 U/mg specific activity with 9 fold purification (Table 1). Affinity chromatography results the final purification of Amy RM44 with 31% yield. As through affinity chromatography purification of enzyme was achieved on single step therefore it replaced the traditional chromatography purification steps. Therefore research has been carried out for the optimization of Affinity chromatography procedure. Several absorbents were available and prepared for the affinity of enzyme. Our selection of insoluble corn starch was based on the utilization of cheap and easily available source for Amy RM44 purification. Similarly various reagents such as dextrin and maltose have been reported for the elution of absorbed enzyme from affinity column (6, 7). Among various eluent tested Amy RM44 was efficiently eluted by dextrin gradient.

Molecular weight of Amy RM44 was found to be 66 kDa as revealed by SDS-PAGE and zymography analysis. Activity gel showed two bands in denaturing PAGE while single band on native PAGE (Figure 1). Results suggested that Amy RM44 might be existed in aggregate form from which an active subunit of 66 kDa was separated under denaturing conditions. Amylase showed different positions on SDS-PAGE and native PAGE has been reported (15). Najafi *et al.*, (16) also found that their purified amylase may be a homodimer therefore showed different molecular weight under denaturing and native PAGE. Generally maltogenic amylases were found in more than one form thus there is a possibility that Amy RM44 would be a maltogenic amylase that was further discussed in the later sections.

*Effect of temperature on Amy RM44 activity and stability*

Optimum temperature of Amy RM44 was determined by assaying purified enzyme activity at various temperatures (Figure 2). Results showed that Amy RM44 was a thermoactive enzyme with optimum temperature at 100 ˚C. It has been observed from the obtained data that Amy RM44 was also highly stable at temperature range 80-100 ˚C as retained around 50% activity at 100 ˚C for 2 h (Figure 3). Amy RM44 could be suitable as most of the industrial processes required amylases that were highly active and stable at 95-100 ˚C for bioconversion of starch (17).

*Effect of pH on Amy RM44 activity*

Optimum pH of Amy RM44 was determined in various buffers by using soluble starch as a substrate. Amy RM44 was active over the pH range 5-11 having optimum activity at pH 5 (Figure 4). Results showed that Amy RM44 has the ability to utilize substrates in both acidic and basic conditions. Amylase stable over broad pH range has been reported (18, 19). Optimum pH of Amy RM44 would be useful in food industry where most of the process required acidic pH (20). However, its 80-90% activity towards basic pH would makes it useful in detergent and textile industries.

*Effect of activators and inhibitors on Amy RM44 activity*

Amy RM44 was examined for its activity in the presence of various metal ions, inhibitors, and chelating agents. According to the results Amy RM44 was resistant to EDTA as it retained 85% activity for 1h under standard assay conditions. Results showed that Amy RM44 also retained 75% and 44% residual activity with PMSF and SDS respectively (Table 2). Metal ions may act as activator or inhibitor of amylase. Our data showed that sodium and calcium neither act as activator nor inhibitor for Amy RM44 activity otherwise Amy RM44 activity was reduced in the presence of mercury. It has been reported that most of the amylases increased their activity in the presence of calcium ions (21). In this case calcium was not the activator of Amy RM44 therefore acts as calcium independent enzyme which is the main concern of starch processing industries.

*Hydrolytic activity of Amy RM44 on various substrates*

Among the substrates tested, Amy RM44 showed hydrolytic activity towards pullulan, dextrin, α-cyclodextrin, β-cyclodextrin, and γ-cyclodextrin along with starch (Table 3). Our results corelated with the mode of action of the maltogenic amylase of *Bacillus licheniformis* (22), *Bacillus stearothermophilus* (23) and *Bacillus thermoalkalophilus* (24). Multisubstrate specificity of Amy RM44 therefore suggested that it may be a maltogenic amylase. 

## Conclusion

There are no reports on the production and purification of amylase from *Bacillus licheniformis* RM44. Findings of this research concluded that *Bacillus licheniformis* RM44 was found to be a thermoactive and thermostable novel amylase producer. Affinity chromatography showed better purification of Amy RM44 to homogeneity. Characteristics of enzyme such as high optimum temperature and thermal stability demonstrate Amy RM44 as a useful candidate for starch industry. Chelator resistant and calcium independent property of Amy RM44 would also be beneficial to the cost of the process. Further, its activity at broad pH range as well as multisubstrate specificity suggests great potential for industrial processes. 
